# Reliability and validity of the Chinese version of the Childhood Trauma Questionnaire-Short Form for inpatients with schizophrenia

**DOI:** 10.1371/journal.pone.0208779

**Published:** 2018-12-13

**Authors:** Wen-Juan Jiang, Bao-Liang Zhong, Lian-Zhong Liu, Yong-Jie Zhou, Xiao-Hua Hu, Yi Li

**Affiliations:** Department of Psychiatry, Affiliated Wuhan Mental Health Center, Tongji Medical College of Huazhong University of Science and Technology, Wuhan, P. R. China; Sapienza University fo Rome, ITALY

## Abstract

**Background:**

The evaluation of childhood trauma is essential for the treatment of schizophrenia. The short form of Childhood Trauma Questionnaire (CTQ-SF) is a widely used measure of the experience of childhood trauma in the general population. Nevertheless, data regarding the psychometric property of CTQ-SF for assessing childhood trauma of patients with schizophrenia are very limited.

**Methods:**

Two hundred Chinese inpatients with schizophrenia completed the Chinese CTQ-SF, the Child Psychological Maltreatment Scale (CPMS), the Impact of Events Scale-Revised (IES-R), and the Dissociative Experiences Scale-II (DES-II). To assess test-retest reliability of the CTQ-SF, all patients completed the CTQ-SF again two weeks later. Concurrent and convergent validity was assessed by analyzing Pearson bivariate correlation coefficients between CTQ-SF and CPMS, IES-R, and DES-II.

**Results:**

The Cronbach’s α coefficient of the Chinese CTQ-SF was 0.81, and the two-week re-test reliability was 0.81 (P<0.01). The criterion-related validity coefficients of CTQ-SF with the CMPS, IES-R and DES-II were 0.61, 0.41, and 0.51, respectively.

**Conclusion:**

The Chinese CTQ-SF has satisfactory psychometric properties to measure childhood abuse or neglect in Chinese inpatients with schizophrenia.

## Introduction

There is convincing evidence that patients with schizophrenia are at elevated risk for childhood trauma, and the childhood trauma is significantly associated with negative health outcomes in this patient population, including severe psychotic symptoms, non-adherence to treatment, and poor psychosocial functions [1–2]. Childhood trauma is an important environmental factor that interacts with genetic predisposition to influence the expression of schizophrenia [3–4]. The role of childhood trauma is thought to be pivotal in the development of schizophrenia: influencing both the manifestation and progression of this disease [5–6]. Some authors have also argued that childhood trauma leads to excessive glucocorticoid production and subsequently causes neurotoxicity to the hippocampus, which, in turn, results in psychotic symptoms in schizophrenia [7–8].

Further evidence from recent studies has demonstrated significant predictive effects of childhood trauma on poor response to treatment with antipsychotics [9] and higher doses of medications, particularly psychotropic medications and mood stabilizers [10]. An increasing literature emphasizes the need for an accurate, early assessment of childhood trauma in schizophrenia and encourages a subsequent trauma-sensitive treatment plan [11].

Routine assessment of childhood trauma and an individualized bio-psycho-social formulation are necessary for personalized treatment of schizophrenia [12]. Therefore, when patients are diagnosed with schizophrenia, they should receive a proper childhood trauma assessment and be offered psychological treatments to address the sequelae of the childhood trauma or abuse. There is a pressing need to address childhood trauma in this patient population, which is required by a broadening range of available treatments [13].

One impediment to the acceptance and implementation of these recommendations for schizophrenia patients is the lack of a psychometrically sound tool. Some instruments have been developed to assess childhood abuse and neglect. The Short Form of Childhood Trauma Questionnaire (CTQ-SF) is one of the most widely used tools to assess the experience of childhood trauma of both general populations and clinical patients and has become a leading measurement in western countries [14,15]. It also has good performance in the validity and reliability of various language versions [16–21]. However, the psychometric literature regarding the CTQ-SF in schizophrenia is sparse. There is a meaningful beginning with the study to confirm the reliability and validity in both outpatients and inpatients with schizophrenia in Korea [22–23]. Due to cultural difference across countries, additional studies are necessary to assess the psychometric property of CTQ-SF in China.

To the best of our knowledge, to date, there have been no data regarding the reliability and validity of the Chinese CTQ for assessing the childhood trauma of patients with schizophrenia. This study explored whether the Chinese version of the CTQ-SF is applicable for the assessment of childhood trauma in Chinese inpatients with schizophrenia.

## Methods

### Ethics statement

The study protocol was reviewed and approved by the Institutional Review Board of Wuhan Mental Health Center. Before participating the study, all subjects were fully informed of the study’s objectives, content, and procedures, and confidentiality and declarations of anonymity had been made. Written consent was obtained from all subjects and their guardians (when necessary) prior to the study. For the compensation of time spent in participating in this study, each patient was given a gift valued at 15 US$.

## Settings and subjects

Subjects were inpatients at Wuhan Mental Health Center, the largest psychiatric specialty hospital in south-central China. A convenient sample of inpatients with schizophrenia was recruited from September 2015 to March 2017. Patients were considered qualified for the study if they 1) met the diagnosis of schizophrenia according to the ICD-10 as assessed by experienced psychiatrists; 2) were clinically stable (total score of Positive and Negative Syndrome Scale [PANSS] ≤40); 3) aged between18 and 40 years; and 4) had an educational attainment of at least primary school. Patients were excluded if they had 1) a severe physical illness, drug abuse/dependence or other psychosis; 2) an intelligence quotient (IQ) score less than 80 or 3) difficulties in understanding questions of the study questionnaire.

## Procedures and measures

Three trained attending psychiatrists screened patients for eligibility, and sought consent from eligible patients to participate in the study. Participants were instructed to independently and anonymously complete the study questionnaire and completed the CTQ-SF again two weeks later.

The study questionnaire consisted of a basic information form and four scales. The basic information form was used to collect data on patients’ demographics (age, sex, education level, and marital status) and clinical characteristics (family history of psychosis, number of previous admissions, and duration of illness). The four scales were described as below:

1) Chinese version of the CTQ-SF [24]

The CTQ is a retrospective self-report scale developed by David Bernstein and colleagues, which has 70 items [25,26]. The CTQ-SF consists of 28 items of the original version of CTQ: five items for each of the five types of neglect or abuse [emotional neglect (EN), physical neglect (PN), emotional abuse (EA), physical abuse (PA), and sex abuse (SA)] and three items for the tendency to minimize/deny. Each item assesses the frequency of trauma experience by using a 5-point Likert-type response, ranging from 1 = never to 5 = very often. The total score of each subscale ranges from 5 to 25. Cut-off scores for separating subjects with none, low, moderate, and severe degrees of trauma for each subscale was reported in an early publication [26]. The Chinese translated version of CTQ-SF, developed by Zhao and colleagues, has been confirmed to exhibit good psychometric properties and cultural equivalence in Chinese population [20,24], which was directly used in our study.

2) Child Psychological Maltreatment Scale (CPMS) [27]

The concurrent validity of the CTQ-SF was evaluated with the 23-item self-report CPMS. CPMS is developed to measure childhood neglect and abuse for adolescents in China and it has five subscales: terrorizing, ignoring, belittling, intermeddling, and corrupting. In this study, we used two subscales of CPMS only: neglect (ignoring and corrupting) and abuse (terrorizing, belittling, and intermeddling). Each item of the CPMS is rated using 5-point Likert-type scale, ranging from 0 = never happen to 4 = very often happen. This scale has an acceptable psychometric property in Chinese students [27,28].

3) The Chinese version of the Impact of Events Scale-Revised (IES-R-C) [29]

The self-report IES-R-C scale measures the symptoms of posttraumatic stress disorder (PTSD) [30]. Because the symptoms of PTSD are more prevalent among individuals who have experienced early trauma and abuse [31], and studies have shown the significant correlations between childhood trauma experiences were significantly correlated with the PTSD symptoms [17, 31–34], the 22-item IES-R-C scale was adopted to assess the convergent validity of the CTQ-SF. The Chinese version of the IES-R has adequate psychometric properties among junior and senior students [29,30].

4) The Chinese version of the Dissociative Experiences Scale-II (DES-II-C) [35]

The DES-II-C was also administered to assess the convergent validity of CTQ-SF, because dissociative experiences frequently occur in patients with early trauma [36,37], and childhood trauma experiences are significantly correlated with dissociative symptoms [21,38–40]. This self-report scale is used to measure the continuum of dissociation, ranging from normal to pathological dissociative symptoms. The DES-II-C has adequate reliability and validity in Chinese undergraduates and inpatients with mental illness [35,41].

## Data analysis

The internal consistency of the CTQ-SF-C was evaluated using Cronbach’s α coefficient. Because scores of the CTQ-SF, CPMS, IES-R-C, and DES-II-C were not normally distributed, Spearman’s correlation coefficients were calculated for assessing test-retest reliability and concurrent and convergent validity. All data were analyzed by using SPSS (version 20.0) for Windows, and statistical significance was set at an alpha level of 0.05 (two-sided) for all tests.

## Results

### Demographic characteristics and prevalence of childhood trauma

A total of 300 inpatients were screened, among whom 243 were eligible for the study but 35 declined to participate. Finally, 208 agreed to participate and 200 completed the study questionnaires.

Demographic characteristics of the 200 subjects are presented in Table 1. The mean age of subjects was 28.3 years (standard deviation [SD] = 5.9). Medians of the duration of schizophrenia illness and total number of previous psychiatric hospitalizations were 60.1 months (range: 1 month–28 years) and 3 (range = 0–39), respectively.

**Table 1:** Characteristic of participants with schizophrenia.

Variables	Total (n = 200)	Male (n = 100)	Female (n = 100)	statistics	P
Age (years)	28.3(5.9)	28.1(5.6)	28.4(6.2)	t = 0.30	0.10
Months of illness	60.1(52.7)	62.0(54.1)	58.3(51.3)	U = 4873.0	0.75
No. of previous hospital admissions	3.0(1.8)	3.2(1.8)	2.7(1.8)	U = 4193.0	0.04
Marital status					
Married	133(66.5)	73(73.0)	60(60.0)	x2 = 3.79	0.05
Unmarried	67(33.5)	27(27)	40(40.0)		
Education					
Below senior high school	69(34.5)	37(37.0)	32(32.0)	x2 = 1.73	0.21
Senior high school	74(37.0)	37(37.0)	21(21.0)		
Bachelor	51(25.5)	28(28.0)	35(35.0)		
Post-graduate	6(3.0)	8(8.0)	12(12.0)		
Family history of psychosis					
Positive	36(18.0)	19(19.0)	17(17.0)	x2 = 0.14	0.71
Negative	164(82.0)	81(81.0)	83(83.0)		

Note: continuous and categorical variables are expressed as mean (standard deviation) and number (%), respectively.

The average total score of CTQ-SF was 43.4 (SD = 13.9). The average total scores of EN, PN, EA, PA, and SA subscales of CTQ-SF were 11.6 (SD = 5.0), 9.3 (SD = 3.6), 8.6 (SD = 3.6), 7.2 (SD = 3.1), and 6.6 (SD = 2.7), respectively. According to recommended cut-off scores for low-to-moderate severity of childhood trauma [26], EA (≤12) was the most common type of trauma (n = 155,77.5%), followed by EN (≤14) (n = 151, 75.5%), PN (≤9) (n = 131, 65.5%), PA (≤9) (n = 95, 47.5%), and SA (≤7) (n = 74,37%), while for moderate-to-severe trauma, PN (≥10) was the most common (n = 31,15.5%), followed by EN (≥15) (n = 19, 9.5%), SA (≥8) (n = 10, 5.0%), PA (≥10) (n = 5, 2.5%), and EA (≥13) (n = 2, 1.0%).

### Internal consistency and re-test reliability

The Cronbach’s α coefficient of the CTQ-SF was 0.81, and the α coefficients for the five subscales ranged from 0.61 (PN) to 0.84 (PA). The two-week test-retest reliability coefficient of the CTQ-SF was 0.81, and corresponding coefficients for the five subscales ranged from 0.58 (SA) to 0.78 (PN). All these correlations were statistically significant (p<0.01) (Table 2).

**Table 2:** Internal consistency and two-week retest reliability of the CTQ-SF among Chinese inpatients with schizophrenia.

	CTQ-PA	CTQ-SA	CTQ-EA	CTQ-EN	CTQ-PN	CTQ total
Cronbach`s α coefficient	0.84	0.83	0.80	0.82	0.61	0.81
Two-week retest reliability	0.65**	0.58**	0.70**	0.70**	0.78**	0.81**

Note: CTQ-SF: The Childhood Trauma Questionnaire-Short Form, PA: physical abuse, SA: sexual abuse, EA: emotional abuse, EN: emotional neglect, PN: physical neglect, EN: emotional neglect.

*P<0.05.

**P<0.01.

### Concurrent and convergent validity

The total CTQ-SF score was significantly correlated with the total CPMS score (r = 0.61). The five subscales of CTQ-SF and the two subscales of CPMS were also significantly correlated (abuse: 0.39–0.64; neglect: 0.22–0.43) (Table 3).

**Table 3:** Correlations of the CTQ-SF and its subscales with CPMS, IES, and DES.

	CTQ total	PA	SA	EA	EN	PN
CPMS-abuse	0.60**	0.45**	0.42**	0.64**	0.42**	0.39**
CPMS-neglect	0.46**	0.22**	0.19**	0.43**	0.39**	0.39**
CPMS-total	0.61**	0.39**	0.36**	0.62**	0.45**	0.43**
IES-R	0.41**	0.26**	0.34**	0.45**	0.30**	0.23**
DES-II	0.51**	0.41**	0.33**	0.49**	0.27**	0.45**

Note: CTQ-SF: The Childhood Trauma Questionnaire-Short Form, PA: physical abuse, SA: sexual abuse, EA: emotional abuse, EN: emotional neglect, PN: physical neglect, CPMS: Child Psychological Maltreatment Scale, IES-R: the Impact of Events Scale-Revised, DES-II: Dissociative Experiences Scale-II. * P<0.05.

** P<0.01.

The total scores of CTQ-SF and its five subscales were all significantly correlated with IES-R-C and the DES-II-C (r = 0.23–0.51) (Table 3).

## Discussion

The cross-cultural and cross-population validation of childhood trauma screening measures in schizophrenia is a necessary and important work for both clinical and research work. Findings from the present study provide the basis for applying this scale in clinical work, for example, the CTQ-SF may be particularly useful for patients with refractory schizophrenia given their nonresponse to antipsychotics: psychotherapy based on childhood trauma experiences provides one potential effective treatment choice [12,13].

Overall, the CTQ-SF had good reliability for Chinese inpatients with schizophrenia in terms of internal consistency and test-retest reliability coefficients. In general, the Cronbach’s α coefficient of a psychometrically sound scale should be greater than 0.7 [42]. The low internal consistency of the PN subscale (α = 0.61) of CTQ-SF is consistent with previous studies [19,23,42,43]. We consider that this phenomenon might be ascribed to the cultural differences in the definition of physical neglect across countries, for example, most Chinese people believe “spare the rod and spoil the child” but most people of western countries do not think so. Compared with a similar study in Korea, our study showed a better test-retest reliability of CTQ-SF [23], indicating the good stability of trauma experiences as measured by CTQ-SF in Chinese inpatients with schizophrenia.

In this study, the good concurrent validity of the CTQ-SF was confirmed by the significant correlation between CPMS and CTQ-SF. The good convergent validity of the CTQ-SF was also proved by the significant correlations between CTQ-SF and IES-R-C and the DES-II-C. Similar to previous reports [36,44,45], this study found that the five types of childhood trauma were all related to PTSD and pathological dissociation symptoms. Moreover, the low-to-moderate correlations between the CPMS neglect subscale and CTQ-SF subscales were consistent with findings from the inpatient sample of the Korean study [23].

Overall, the prevalence pattern of the five types of trauma experiences in Chinese patients with schizophrenia as measured by CTQ-SF is similar to some but not all previous reports [16,22,23,46–48]; the discrepancy might be explained by a variety of clinical factors, including the mean age of the patient sample and clinical settings (i.e., inpatient vs. outpatient).

This study has several limitations. First, participants were 200 inpatients of one large psychiatric hospital only, limiting the generalizability of the study findings. More studies are warranted to further examine the psychometric property with samples of patients from both outpatient and inpatient clinical settings, as well as patients from other institutions. Second, strictly speaking, the CPMS has no the construct of SA, therefore using CPMS to examine the concurrent validity of SA of the CTQ-SF might be problematic. Nevertheless, the CPMS was still adopted for this study because it is a widely used instrument for the assessment of childhood neglect and abuse in China, despite not specific to SA.

In summary, this study confirmed adequate reliability and validity of the CTQ-SF for assessing childhood trauma experiences in patients with schizophrenia in in Chinese inpatient settings. More studies are warranted to address the low internal consistency of PA subscale of CTQ-SF, as well as the definition of childhood trauma, particularly regarding neglect, in China’s unique culture. A precise and conformity definition would help develop a more culturally relevant version of the CTQ-SF, which is particularly necessary in psychiatric clinical settings of China.
